# Intercourse as Affecting Uterine Diseases, and Their Treatment

**Published:** 1861-09

**Authors:** Augustus K. Gardner

**Affiliations:** Professor of Clinical Midwifery and Diseases of Women


### INTERCOURSE AS AFFECTING UTERINE DISEASES, AND THEIR TREATMENT.

BY AUGUSTUS K. GARDNER, M. D.,

*Professor of Clinical Midwifery and Diseases of Women.*

This case is one of an ordinary character; some uterine congestion, hypertrophy of the cervix, etc. It is not especially interesting in itself, but in connection with it, the question is raised, *Whether sexual intercourse is or is not to be avoided during the treatment*, or in order to effect a cure? Very frequently during my course of lectures this winter, I have alluded to the fact that various diseases of the female organs were aggravated, and produced by the sexual congress; yet still the subject has been but imperfectly presented. This morning, I will still further elucidate it. Coition is so important a physiological function, and it is so difficult for the young practitioner to learn respecting the ills which arise from its abuse, either from actual experience of their treatment, or by their study in scientific works which allude but very imperfectly to them, that the theme may well deserve your serious and most studious attention, as it may not only save embarrassment, but also enable you the more easily to recognize and properly treat the many ills which result from overaction in this direction.

The earliest period to which the physician is summoned on account of physical injury happening to the female is immediately after the consummation of the marriage. It is too often the case that, inspired with the idea that every man must in like circumstances perform his part manfully, that the husband does it brutally, and the immediate result is a *laceration of the hymen fourchette* and soft parts, followed by hæmorrhage, sometimes so profuse and uncontrollable as to demand the physician's attention. Usually, this hæmorrhage will be easily arrested by cold water, ice to the vulva, or what is less dangerous, pressure, or a pledget of lint wet in solution of the liquor perchloride or sulphate of iron, more or less diluted, and pressed firmly upon the bleeding part; sometimes a little arterial vessel is ruptured, and it may be necessary to twist it with a torsion forceps. In rarer instances, it is requisite to take up the artery; while generally, holding the tissue pressed firmly between the thumb and fingers for ten or fifteen minutes, will suffice to stop the flow, and by rest and cold applications it will generally be prevented from recommencing.

Sometimes, instead of any laceration, with rupture of any vessel, the parts are much bruised with *ecchymosis and extravasation into the labia majora*, which are consequently much swollen and painful. This condition is best treated by cataplasmata or cold applications over the parts; and a few days only are required, with rest and light diet, if the inflammation be sufficient to demand constitutional treatment, to effect resolution, absorption, and cure. Sometimes, however, this is not the case, and the parts go on to suppuration; an abscess forms, which, if not opened, may spread through the loose cellular tissue and become very large, and greatly destroy the parts. Occasionally, even more serious results, from the supervention of erysipelas, may be effected; and I have known an abscess to be thus formed in the labium, which, after breaking and discharging its contents, a day or two after the ulceration had spread so as to destroy the artery, which bled so profusely in spite of all the efforts of most skillful surgeons to arrest it, as quite to threaten the life of the patient; and it was not until the abscess was completely laid open, the artery found and tied, by a long, serious, and painful operation, that the hæmorrhage was finally stopped.

The effect of coitus is very frequently seen in the *inflammation of the labio-vaginal gland*, sometimes called Hugier's gland, from its recent discoverer and pathologist. This is one of the commonest forms of acute inflammatory disease of these parts, and is caused not only by coitus, but also by the friction arising in the course of horseback riding, particularly in those unaccustomed to this exercise, and more especially when this excitation is taken during the period of the menstrual congestion. The abortive treatment by cold and astringent applications and leeches, or failing in that, by cataplasmata and the subsequent opening of the abscess, it is not necessary to enlarge upon here.

*Vaginitis* is also the direct result of excessive coition. This complaint does not materially differ, as I have described to you, from gonorrhœa, unless it be that the latter is more virulent. Indeed, I am very doubtful if gonorrhœa should be considered a specific disease; certainly I have seen cases of vaginitis not different in any respect from gonorrhœa after a connection of unquestionable purity; and it is generally recognized that urethritis in the male is often produced without an impure connection, being the result of uterine leucorrhœa of an acrid character. It is considered by some that the presence of urethritis complicating vaginitis is a sign of the specific character of the complaint; yet this, too, I am convinced, is incorrect, for reasons similar to those just enunciated.

Vaginitis is unquestionably the product of excessive sexual intercourse, and we find it perhaps more frequently in the wives of young, sanguine clergymen, and other men of like continent habits. Unlike the majority of the young men of cities, at least at the present day, their youth is spent in abstinence and imagination. Marriage with them is a rite which justifies the freest and fullest indulgence of their pent-up passions, and the consequence is that they are apt to go to such excess that disease is the result. Moderation in the indulgence of all the appetites is absolutely requisite to avoid disease.

So far as the treatment is concerned, antiphlogistics, rest, and mild tropical astringents, a grain or two of the nitrate of silver to an ounce of water, often repeated, into the vagina, and an unstimulating diet, is all that is requisite. For a local application I have found no injection more efficacious than the following formulæ :

℞.—Zinci Chloridi, ʒ ij.

Aquæ Puræ, ʒ iij. M.

Or, what is about the same,

℞.—(Squibb's) Liquor Zinci Chloridi.
Eight or ten drops of either to a tumbler of water.

Not unless the urethra is implicated is any medicine by the mouth necessary. If so, copaiba, cubebs, potassæ, etc., to act specifically and modify the acridity of the urine, will be found requisite.

Coitus does not, in the normal condition of the uterus, produce disease of the surface of the cervix. It, however, if very frequent, brutal, or in consequence of a too long virile organ, will produce congestion of the organ; then, from its inflamed condition, abrasion of the mucous membrane; from a consequent hyperæmia, hypertrophy, and increased weight, consequent prolapsus, and a whole train of depending symptoms. Where these symptoms exist, if acute disease continues, coitus is inconsistent with cure. When, however, we have chronic hypertrophy of the cervix, endometritis, as in the case now before us, I find that moderately frequent intercourse, entered into without an inordinate sensuality, is beneficial, and so far from being the cause of injury, acts as a direct stimulus to the parts, unloading the congested and turgid glands, and by exciting them to a more profuse secretion, tends to reduce the hyperæmia of the organ.

When, however, there is simple abrasion of the cervix, unaccompanied by hypertrophy and thickening of the parts, or even deeper ulcerative disease, which is very rare, intercourse will generally be found productive of bad results. The glandule lining the neck not participating in the disease, no benefit will accrue from their stimulation and unloading; and very frequently disease may be lighted up in them and spread either as an acute or chronic disease, with that facility of "propagation by neighborhood" so marked in uterine diseases, upon which I have already often enlarged.

Where hæmorrhage follows immediately after coition, with or without pain, there is always some disease present. It does not generally proceed from the just described ulcerations, unless they be of the *fungous variety*, regarding which you have been instructed; but it is usually an evidence of more serious disease, perhaps of a small mucous polypus, or a fibroid, either pediculated or submucous, or of a cancerous or phagædenic ulcer, to which appropriate treatment should be directed. The hæmorrhage produced by coition from a mucous polypus not larger than a pea, is sometimes exceedingly persistent and debiliating.

Coition in those suffering from endometritis is not necessarily in all instances injurious, but it is apt to be attended by sudden and most intense spasms of pain simulating colic, which comes on not unfrequently in the course of the act. I have one case in my memory where I administered a tablespoonful of the Tinct. Opii without relief, and was obliged to give additional doses of the same narcotic before the violence of this intense spasm was assuaged. Sometimes these pains come on at other periods, especially after the sudden suppression of the menses. These occur very commonly among prostitutes, and are sometimes called *colica scortorum*; and their pathological character not being well understood, they are variously considered to be peritonitis, inflammation of the bowels, sometimes more nearly as metritis; but in almost all these cases are improperly treated by mercurials, from which I have seen many uselessly salivated. The temporary treatment is opiates and hot fomentations, and subsequently leeches, or scarification of the neck, intra-uterine applications of styptic unguents, and generally abstinence from sexual intercourse. The latter is, however, the most difficult prescription to enforce among the class in whom it is so apt to occur, already referred to.

In the pregnant, coition is often to be entirely prohibited, especially in those who have had previous abortions. I have elsewhere stated that for this falling of immature fruit there is always a cause. Often it is owing to ulceration of the os, fissures of the cervix, which are separated by the development of the womb and the absorption of the neck into its substance. Coition starts up a slight bleeding, which is speedily increased, till it becomes so augmented that an abortion is the almost inevitable result. *Obsta principiis* is the rule, and abstinence from coition and the freedom from all uterine irritation is the method of enforcing it.

So, too, in the cases of so-called "irritable uterus," or in those women "who have a tendency to abort at certain periods," but where there is always some certain and appreciable disease, be it as above mentioned, or endometritis, little polypi, spinal irritation, etc., coition is absolutely to be avoided until the usual period for abortion has been assuredly passed, and resumed only with the greatest care. In fact, excessive intercourse will of itself produce abortion, as certainly and for the same reason as the water douche, by simple uterine irritation. This is, in my opinion, the reason why prostitutes so rarely become mothers. It has been supposed by some to be the mixing of various semen, etc.; but I believe it to be simply and solely because the uterus is not allowed the rest indispensable to form the *nidus* requisite for the ovum, but, constantly irritated, throws it off in the earliest stage. Of course a career of prostitution eventually so disorders the generative apparatus that they are rendered incapable of fulfilling their functions under any auspices.

Finally, to conclude this hasty *résumé* of the hygienic proprieties of coition, after delivery, this act should be renewed with great circumspection, and not until a proper period of time has elapsed. Not unfrequently are the sexual relations re-entered upon with a haste savoring more of bestiality than of creatures endowed with reason and sensibilities. The period cannot be determined by days, but time should elapse sufficient for the entire and complete cessation of all profluvia, for the resolution of the uterus, for the restoration of the vagina and perineum to their normal tone and contractibility; operations which will rarely be completely effected in less than a month, and which usually exceed this period. Indeed, we not unfrequently find the bloody flux continuing double this period. The poetic exaggerations of Michelet, in regard to the "internal, constant wound" with which woman suffer, is in this respect more worthy of consideration than in any other.

I have thus hastily strung together, and presented with some attempt at sequence, views which have been enunciated throughout the course. Their careful consideration will, I am sure, save you from much anxiety in your future career. They should be considered not as a prurient theme for a vicious imagination, but simply and solely as an investigation of great physiological and pathological laws. Such contemplations of any function in God's creation elevate and refine the man, while the morbid idealism by the depraved mind but degrades the thinker, and dishonors the Creator of all things.—*Am. Med. Times.*

